# Do Participants in Genome Sequencing Studies of Psychiatric Disorders Wish to Be Informed of Their Results? A Survey Study

**DOI:** 10.1371/journal.pone.0101111

**Published:** 2014-07-01

**Authors:** Elise T. Bui, Natalie K. Anderson, Layla Kassem, Francis J. McMahon

**Affiliations:** Human Genetics Branch, National Institute of Mental Health, National Institutes of Health, Bethesda, Maryland, United States of America; Universite de Montreal, Canada

## Abstract

**Objective:**

As large-scale genome sequencing technology advances, concerns surrounding the reporting of individual findings to study volunteers have grown and fueled controversy. This is especially true in mental health research, where the clinical importance of sequencing results is particularly unclear. The ethical, legal, and social issues are being widely debated, but less is known about the attitudes of actual study volunteers toward sequencing studies or what they wish to learn about their DNA sequence and its health implications. This study provides information on psychiatric research volunteers’ attitudes, beliefs, and concerns with respect to participation in DNA sequencing studies and reporting of individual results.

**Method:**

We conducted a pilot study using a questionnaire that we developed to assess what information volunteers in an ongoing family study of bipolar disorder would like to receive if they underwent genome sequencing, what they would do with that information, and what concerns they may have.

**Results:**

Almost all of the respondents were willing to participate in genome sequencing. Most respondents wished to be informed about all their health-related genetic risks, including risks for diseases without known prevention or treatment. However, few respondents felt well informed about the nature of genome sequencing or its implications for their health, insurability, or offspring.

**Conclusions:**

Despite generally positive attitudes toward genome sequencing among study volunteers, most are not fully aware of the special issues raised by genome sequencing. The attitudes of study volunteers should be considered in the debate about the reporting of individual findings from genome sequencing.

## Introduction

Large-scale (exome and whole-genome) sequencing has become an essential tool in the study of human genetics and disease, revealing the genome in far greater detail than previously possible. As the field of human genetics progresses toward the whole-genome sequencing era, researchers need to face careful ethical considerations regarding the vast amount of data revealed about each study participant [1]. This is especially true in mental health research, where the clinical importance of sequencing results is particularly unclear, and study participants often cope with psychiatric illness that impacts mood, cognition, or judgment. A number of bioethicists assert that *aggregate* study results should be released to clinical trial participants [2]–[4]. Others argue that *individual* genetic results should be offered to study participants, but this raises concerns [5]. While bioethicists and researchers are debating these topics, few data have been collected about the attitudes of actual study volunteers regarding these issues.

When the entire genome is interrogated, discovering genetic information that goes beyond the aim of a study– an incidental finding– is inevitable. Many ethicists agree that researchers have an ethical obligation to report to participants genetic variants that are clinically significant [6]–[9], but opinions vary as to what “clinically significant” really means. Working group guidelines from the National Heart, Lung, and Blood Institute (NHLBI) indicated that return of results *should* occur if the genetic finding is established, actionable, and valid [10]. The American College of Medical Genetics (ACMG) recently issued a similar set of recommendations aimed at sequencing in clinical – rather than research – settings, along with a list of genetic mutations that constitute “…a ‘minimum list’ of incidental findings to report from clinical sequencing” [11]. The reaction varied from praise to outrage [12], [13].

Beskow and Burke assert that researchers have a “duty to rescue” volunteers if they uncover genetic information that “indicates a higher probability of a serious condition for which an effective intervention is readily available” [2]. However, a subsequent interview study of investigators found resistance to the idea of a “duty” to disclose results, because such a disclosure blurs the distinction between research and clinical practice. Of course, volunteers may explicitly *not* want to know details about their genome. While many argue this desire not to know must be respected [6], [7], [14]–[16], ACMG originally recommended reporting results systematically without referencing patient preferences [11]. However, in response to concerns about protecting patient autonomy, the ACMG published a follow-up paper underscoring their respect for patient autonomy and for clinicians’ non-disclosure of unwanted information – provided that they ensure the patient is well informed about this decision and document the reasons for non-disclosure [13]. The ACMG has acknowledged that their recommendations are a “work in progress” [11], [13], and most recently has voted to give patients a choice to opt-out of learning incidental information [17]. Interestingly, one recent focus group study demonstrated a consensus among genetics professionals, the general public, and parents of patients with genetic disorders that participants should be primarily responsible for tracking developments in research and recontacting researchers or clinicians if they so choose [16].

While opinions on the return of genetic results vary greatly among experts and the public, the attitudes of actual genetic study volunteers have not been fully examined, although this literature is growing. In one recent study, Facio et al found that all 311 individuals enrolled in a genetic study indicated that they would want to receive their individual results, especially if the results pointed to a preventable condition or one that could be passed to offspring [18]. In another study with participants whose families were affected with the autosomal recessive disorder Miller’s Syndrome, one out of the two families was interested in learning their sequencing results [19]. One interview study of patients attending genetics clinics (along with their spouses and parents) revealed limited enthusiasm for clinical genetic testing. There was more interest in genetic testing that could provide a diagnosis, in contrast to tests that only assess risk in pre-symptomatic patients [20].

To our knowledge, no study to date has assessed attitudes of psychiatric study volunteers or their relatives toward genome sequencing. Two previous studies, before the genome sequencing era, suggested that participants with psychiatric disorders had positive attitudes toward genetic testing, but the return of unanticipated results was not addressed [21], [22]. It is important to explore the attitudes of these volunteers because unique participant characteristics (e.g., emotional states, family history of disease) are factors that may influence attitudes towards genetic results, such as behavior change after receiving results [23].

Here we used a questionnaire administered by a trained interviewer to gain some insight into affected and unaffected volunteers’ attitudes toward participating in and receiving health-related results from a genome sequencing study. We hypothesized that the familiality, impairment, and stigma associated with bipolar disorder might negatively affect participants’ attitudes toward genome sequencing and receiving information regarding health-related diseases. Instead, the results showed that a large majority of respondents were interested in participating in genome sequencing studies and wished to be informed about all their health-related genetic risks, even though most respondents did not feel well informed about genome sequencing or its implications for their health, insurability, or offspring.

## Methods

### Sample

As part of the NIMH Bipolar Disorder Genetics Initiative, a large multi-site study, detailed clinical data and blood samples were collected on families ascertained through a sibling pair affected with bipolar type I or schizoaffective bipolar disorder. For this survey we re-contacted (by phone) the 73 volunteers recruited at the NIMH Intramural Program site between 2009 and 2010. All study participants were aware of the nature of the orginal study, and that this survey was hypothetical and not directly related to psychiatric conditions.

Of 73 study volunteers we recontacted, 58 (79%) agreed to participate in the survey, which was administered over the telephone by a single trained interviewer who wrote what the participant was saying as it was being said. Thirty-three (57%) of the respondents were affected by a psychiatric disorder (bipolar type I, bipolar type II, schizoaffective bipolar, recurring major depression), and 22 (38%) were unaffected family members. Three individuals whose diagnostic status was unknown were not included in the affected versus unaffected comparisons. Respondents were from many different regions of the continental United States, since original recruitment was not restricted by geography. The respondents were broadly representative of our overall study sample: Mean age was 49 years (range 23–72 years). 69% female, and 97% self-identified as white. The median level of education was 16 years, equivalent to a bachelor’s degree (range: eighth grade to post-graduate; see Table 1).

**Table 1:** Participant characteristics.

Category	Subcategory	N = (%)^2^
Sample Contact	Total contacted	73
	Participated	58 (79%)
Affected Status^1^	Affected with Major Mood Disorder	33 (57%)
	Unaffected Family Member	22 (38%)
	Unknown	3 (5%)
Gender^1^	Male	18 (31%)
	Female	40 (69%)
Marital Status^1^	Married	35 (60%)
	Single/Never married	13 (22%)
	Separated/Divorced/Cohabitating	10 (17%)
Ethnicity^1^	Caucasian	56 (97%)
	African American	2 (3%)

1 Denominator is 58 within each category.

2 Rounded to nearest whole percent.

### Ethics and Informed Consent

This study was approved by the Central Nervous System (CNS) Institutional Review Board (IRB) of the National Institutes of Health Intramural Research Program (NIH-IRP). Written informed consent, including consent to be re-contacted for follow-up interviews, was obtained from the volunteers at initial enrollment. At the time we conducted this survey, we had not yet offered sequencing to study participants and had not articulated any policy on return of results.

### Instrument

We developed a 22-question multiple choice and free-response questionnaire designed to assess 5 broad areas of information: demographics, beliefs regarding genome sequencing, what participants would want to know, what they would do with the information, and privacy concerns (The Genome Sequencing Attitudes Survey, GSAS; Survey S1). The questions were intended to assess attitudes toward receipt of genetic results for any health condition, not specifically psychiatric disorders. The nature of the results (targeted or incidental) was not specified to the participants. The examples given to participants for potential findings they could receive were risk for cancer, heart disease, Alzheimer’s disease, Huntington’s disease, and diabetes.

### Statistics

Summary statistics, 95% confidence intervals, and Pearson correlations (2-tailed) were computed using SPSS V15.0. Fisher’s Exact tests (FET) were used to compare responses according to gender, age, level of education, active practice of religion, and parent status for each survey question. Chi-square tests with Yates’ continuity correction were used to compare responses of affected individuals and unaffected family members for each survey question. Significance is reported at the 2-tailed p<0.05 level.

## Results

### Interest in sequencing

Almost all (97%; 95% CI 92–100%) of the study volunteers who responded to the survey stated they would be interested in having their genomes sequenced. When respondents were asked about reasons for their interest in participating in a sequencing study, the most common reason cited was “helping to further science” (cited by 48% of respondents; 95% CI 35–61%). Other common reasons included prevention of illness or development of better treatments (38%; 95% CI 25–51%), and helping family members (33%; 95% CI 20–45%).

Knowledge about genome sequencing technology was highly variable among our respondents. Only 34% (95% CI 22–47%) reported having knowledge of genome sequencing technology. Affected individuals were significantly more likely to report having knowledge of genome sequencing technology than their unaffected family members (p = 0.030). Cited sources of this knowledge included the news media (30%; 95% CI 8–52%), classroom or school (35%; 95% CI 12–58%), books (25%; 95% CI 14–46%), and television (25%; 95% CI 14–46%). Respondents who were over the median age of 47 were more likely to report gaining this knowledge from news media (FET p = 0.050).

### Desired information and its potential use

**Figure 1:**
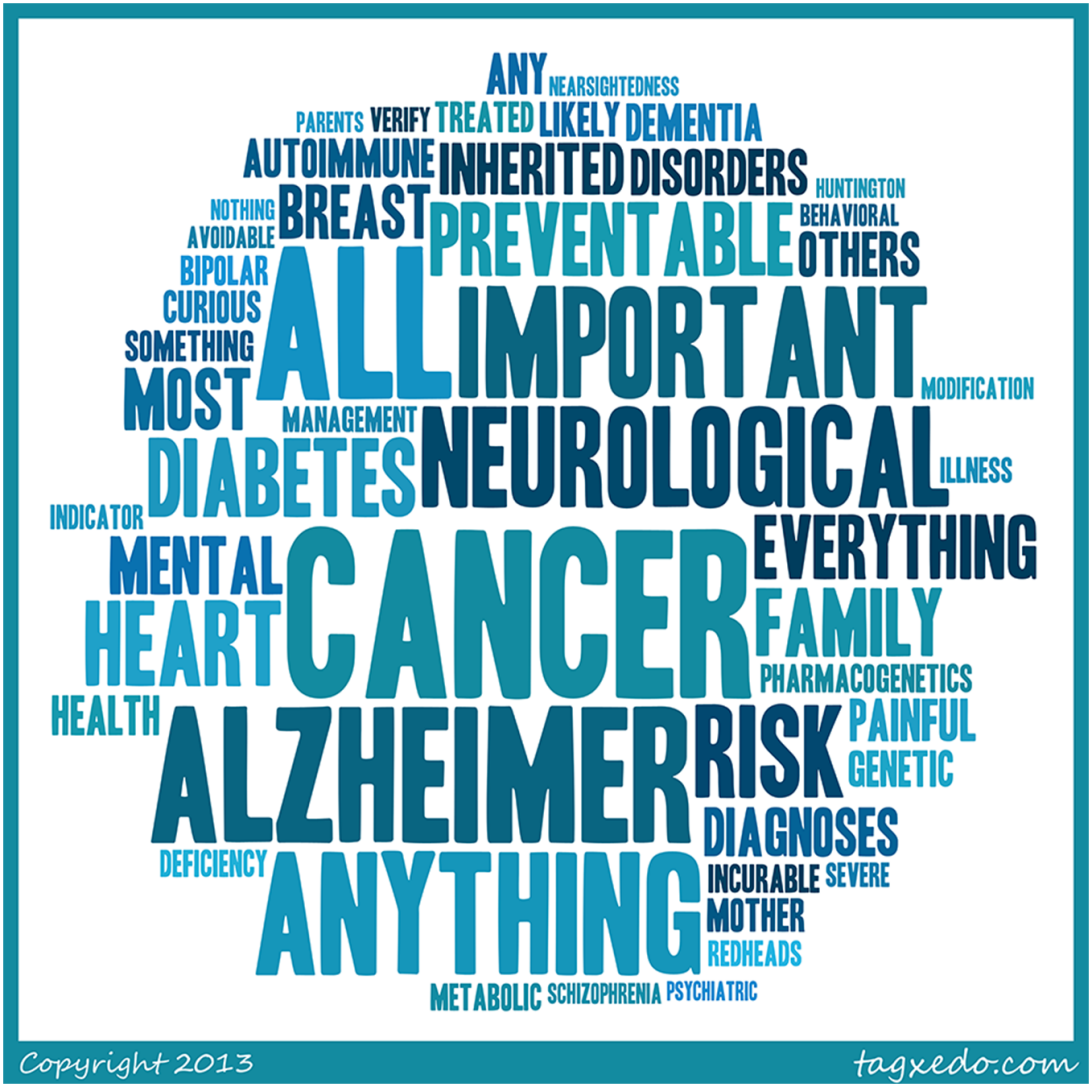
What would be the most important things to know? Figure 1 illustrates participants’ responses to the survey question, “What would be the most important [health information] to know?” in a word cloud, with size directly related to the commonality of the word used in the responses. The word cloud was created using online software at www.tagxedo.com. We entered the text from the participants’ responses free responses. For clarity, we removed common everyday words, combined related words, and set the emphasis to 80%, the maximum word count to 50, tightness to 100%, color variation to 50%, and spread frequency to 20. We edited the style and format for legibility.

Ninety seven percent of respondents wanted to be informed of any genome sequencing information that could have implications for their health when given a binary (yes/no) response choice. When they had the option to qualify this answer in the follow up question, 83% (95% CI 73–96%) still wanted to know about results with “any health implications,” regardless of whether the risk could be modified by prevention or treatment (Table 2). A small group of respondents (10%, 95% CI 2–18%) wanted to be informed only about preventable disorders. An even smaller group (5%; 95% CI 0–11%) wanted to know only about risks that could affect their relatives. The respondents most commonly reported cancer, Alzheimer’s disease, and/or information on all diseases as the most important information to potentially learn (Figure 1).

**Table 2:** Highlighted survey responses.

Question	Response Choices	Number of Respondents^1^ (%)^2^
What sequencing information would you like to know?	Anything that could have health implications	48 (83%)
	Only things preventable or treatable	6 (10%)
	Only things that are very likely to happen	0 (0%)
	Only things that might affect children/grandchildren	3 (5%)
What would you do with this information? *(Multiple answers possible)*	Nothing	1 (2%)
	Talk to doctor	48 (84%)
	Talk to family members	36 (63%)
	Talk to a scientist/geneticist	20 (35%)
Would you change your behavior based on this information?	Yes	28 (49%)
	Maybe	25 (43%)
	No	3 (5%)
	Don’t know	1 (2%)

1 Denominator may not equal 58 due to missing responses or multiple answers possible.

2 Rounded to nearest whole percent.

Few respondents wanted to keep this information to themselves. Eight-four percent (95% CI 74–94) reported that they would discuss their individual results with their primary care physicians. In addition, 63% (95% CI 50–76%) of respondents reported that they would share their information with family members. Only 35% (95% CI 22–48%) reported that they would speak to a scientist or geneticist about their results (Table 2).

Respondents varied in their judgment of what they would do with their sequencing results. Most would consider making a lifestyle change: 49% (95% CI 35–62%) of respondents stated that they would make such a change, while 43% (95% CI 30–56%) said “maybe” and 5% (95% CI 0–11%) said “no.” Responses were not significantly related to age group, gender, marital status, or education level. Typical responses to the open-ended part of this question included diet and exercise changes. Several participants stated that they already have healthy lifestyles and behaviors.

Forty-five percent (95% CI 31–58%) of respondents indicated that knowledge regarding their risk for developing particular diseases would be likely to influence their reproductive decisions, 40% (95% CI 27–53%) indicated that having this knowledge would not influence their reproductive decisions, and 16% (95% CI 6–25%) were unsure. Many respondents indicated that adverse genetic information would “probably” or “seriously” impact their decision to have biological offspring, but some suggested that this decision “depends” on the severity of the risk for offspring.

### Privacy and research ethics

Eighty-nine percent (95% CI 81–98%) of respondents felt their relatives should be told about any risks revealed by genome sequencing, but there was some spread of opinion as to who should communicate this information. Most (85%, 95% CI 75–95%) stated that they should personally be responsible for informing their relatives at their own discretion, while 27% (95% CI 14–40%) stated that researchers should be at least partially responsible for informing family members of potential risk.

Most respondents favored making their anonymized sequence data available for researchers studying other conditions. Ninety-six percent (95% CI 91–100%) of respondents reported that they would agree to their anonymized genome sequence being shared with other researchers. Eight-eight percent (95% CI 77–98%) of respondents stated that they would even agree to allowing their genetic information to be made publicly available, for example, on the Internet, as long as it was anonymous. Open-ended responses generally emphasized confidentiality and anonymity in the service of research to help prevention and awareness.

Even though most respondents supported data sharing across research studies, 39% (95% CI 26–52%) had some concerns about others accessing their genetic information. In the open-ended portion of this question, respondents typically reported they were concerned about how their data would be used by pharmaceutical companies, the government, and insurance companies. This particular concern was driven by respondents’ experiences with insurance companies, and the difficulties they perceived attempting to obtain health insurance with a pre-existing mental illness. When queried about the issue of data access, half (50%; 95% CI 37–63%) stated that they were concerned about genetic discrimination, while the other half (50%; 95% CI 37–63%) were not at all concerned about this.

### How should findings be communicated to participants in genome sequencing studies?

Respondents differed as to the preferred form in which any reportable findings should be communicated. When given the opportunity to select multiple forms of communication, half (51%; 95% CI 37–64%) indicated that they would want to receive the information in a letter, 37% (95% CI 26–52%) selected the telephone, and 25% (95% CI 13–36%) selected email. Only a minority of respondents chose to receive their findings through a health professional: 23% (95% CI 12–34%) through a genetic counselor and 14% (95% CI 5–23%) through their physician. Males were significantly more likely to select email as a preferred communication method (FET p = 0.025). Respondents with a higher level of education were more likely to want to be informed by a genetic counselor (FET p = .023).

## Discussion

To our knowledge, this is the first survey of attitudes toward genome sequencing among actual volunteers in a psychiatric study. While respondents differed in their attitudes about a number of important issues, our findings show that the vast majority are interested in participating in genome sequencing studies, want to be informed about all health-related results, and would allow sharing of their anonymized data among researchers. There were few differences in attitudes between participants affected with a mood disorder and their unaffected family members.

Only 34% of our participants claimed to have knowledge of genetic sequencing technology. However, focus group studies suggest that the general public may be able to understand the nature of genetic research and differentiate among different examples of results they could receive – as they seemed to do in the present study – even if their technical knowledge is limited [18], [24]–[27]. A recent survey of genetic study volunteers found that an educational session with a genetic counselor had no impact on participants’ attitudes toward receiving individual results [17]. Increased knowledge of the limitations on interpretation and usefulness of potential incidental findings also had no effect on participants’ expectations of genetic research or on their desire to receive results [18]. One notable finding in our study was that the affected individuals were significantly more likely to report having knowledge of genetic sequencing technology. This could reflect a heightened motivation to understand their disease.

Our results support the previous findings that volunteers consider information about potential risk factors useful for healthcare and reproductive decisions [18], [26], even though only about half of our respondents reported that they would definitely change their behavior. The REVEAL study on Alzheimer’s disease demonstrated that receiving both targeted and incidental high-risk genetic results led to positive behavior changes in diet, exercise, stress management, and supplement use [23], [28]. More research would be needed to assess if participants underestimated how much they would change their behavior if the receipt of this health information were not hypothetical.

A large majority of respondents in this study stated they would discuss their genetic sequencing results with their primary care physician. Unfortunately, most physicians lack the necessary knowledge and training to help patients interpret genome sequencing results [8], [14]. This highlights an important gap between patient expectations and physicians’ expertise that calls for improved physician education in genetics.

The receipt of genetic information in the mail was the most commonly selected option in our study. Mail may be less costly and time-consuming for all involved, but could lead to the misinterpretation of results. We believe that study participants who opt to receive sequencing results by mail should be provided with contact information for additional counseling and care, as highlighted in a recent focus group study [27]. In our study, the typical reasons participants cited for participating in a genetic study were apparently altruistic, while public attitudes toward potential participation appear to be less so [24], [27] (altruism may well be different when relatives are involved). This highlights a key difference between patients and families affected with a heritable illness on the one hand, and the general public on the other. Another previous study showed that 70% of genetic study participants with mental illnesses believed that awareness of the heritability of psychiatric disorders would help reduce public stigma, although individuals with bipolar disorder were less hopeful about reducing stigma than those with other mental illnesses [21]. Future research is needed to examine how type of illness and family history of illness affect attitudes of genetic study volunteers toward participating and receiving their results.

This study mirrors those of Facio et al and others suggesting that volunteers in genetic studies express a more positive attitude than many researchers and ethicists toward the return of individual results [14], [18]. Most of our respondents wanted to be informed about risks of diseases even without known prevention or treatment. This desire is at odds with the recommendations of a the NHLBI Consensus Panel [14] and the opinions expressed by many investigators [1]. On the other hand, studies of patients and family members in genetics clinics showed less enthusiasm for receiving information for health-related problems from which they did not currently suffer [19], [20], potentially highlighting another difference between members of the general public and families affected with an inherited disease. Such a divergence in attitudes among professionals, and the potential participants themselves, may foretell substantial difficulties in meeting all the expectations of participants in future genome sequencing studies. The recent proposal that study participants choose to track research developments themselves is an intriguing potential solution [16].

Our participants were not given the opportunity to express concern about the psychological impact of learning genome sequencing results. A recent study highlighted concerns for emotional and psychosocial burdens among genetic study participants [20]. On the other hand, as Christensen and Green (2013) discuss [23], normal individuals [28] and mildly cognitively impaired individuals [29] who found out they were at a high risk for Alzheimer’s showed no persistent negative psychological consequences after receiving these results. This finding supports the view that disclosure of incidental findings may not be harmful to study participants.

There were several limitations to our study. The sample size is relatively small, but the 95% confidence intervals demonstrated very large majorities for most questions, so it is unlikely that a larger sample would alter the main findings substantially. The sample is not representative of the general population and consisted entirely of volunteers in an ongoing genetic study, who may be more likely to view genome sequencing in a favorable light. The attitudes of genetic study volunteers are particularly relevant, since they are the most likely individuals to be recruited for genome sequencing studies. The sample comprised patients with bipolar disorder and their relatives, and thus may not represent participants in studies of other inherited illnesses. However, the respondent sample is quite representative of our study volunteers, many of whom are good candidates for genome sequencing studies. The GSAS questionnaire consists entirely of hypothetical questions, and respondents’ answers may not reflect their actual thoughts and behavior if they were provided with real genome sequencing results. Despite these limitations, our findings provide a glimpse of actual study volunteers’ expectations. This can inform the ways in which researchers address the return of sequencing results in future studies.

The attitudes of study volunteers toward genome sequencing are a fundamental component in the ongoing debate over the reporting of individual results to participants. In light of the greater than expected interest among our study participants in learning their sequencing results, there is an urgent need to educate study volunteers, physicians, and other health care professionals about genome sequencing and the use of genomic information in diagnostic and treatment decisions.

## Supporting Information

Survey S1 **Genome sequencing attitudes survey.** Supporting Information File 1 is the “Genome Sequencing Attitudes Survey” (GSAS) that was created and administered in this study.
(PDF)
