# Supplementary material for: Do Participants in Genome Sequencing Studies of Psychiatric Disorders Wish to Be Informed of Their Results? A Survey Study
Source: PLoS One. 2014 Jul 1;9(7):e101111. doi: 10.1371/journal.pone.0101111 (PMC4077756; doi:10.1371/journal.pone.0101111)
Supplement: Survey S1 — Genome sequencing attitudes survey. Supporting Information File 1 is the “Genome Sequencing Attitudes Survey” (GSAS) that was created and administered in this study. (PDF) [file pone.0101111.s001.pdf]

Individual ID: ___________________________

Family ID: ______________________________

Date Completed:________________________

Genome Sequencing Attitudes Survey

Demographics

A. Date of birth: ______________________
B. Gender: (Please Circle) M(1) F(2)
C. Ethnicity/Race _____________________ USE SAME 2-QUESTION SYSTEM AS DIGS FACE
D. Level of education in years __________
E. Profession ________________________USE SAME SCALE AS DIGS
F. Marital Status
    1. Single/Never Married
    2. Married
    3. Separated
    4. Divorced
    5. Widowed
    6. Domestic Partner
G. Number of children ______
H. Religion ________________
   Are you current observant/practicing? Y (1) / N (0)

Beliefs/Behaviors

If we could develop a test that would look at all of your genes and compare them to those of others, in order to better understand how genes might play a role in health and disease…

I. Would you be interested in participating?

1	2	3	4	5
Not at all	Probably not	Somewhat	Probably Yes	Very

J. If yes, what would be your reasons for participating? (Free response)
    1. Curiosity
    2. Contributing to science
    3. Gaining self-knowledge
    4. Prevention/treatment
    5. Concern for family members
    6. Other – specify ____
    ____

____

K. If no, what would be your reasons for not participating?
   1. Lack of interest
   2. Worried how information might be used by others
   3. Worried how the information might affect me
   4. Worried what the information might mean for my relatives
   5. Other - Specify

   ____

   ____

L. Do you have any knowledge about genome sequencing technology?

   Yes (1) **No (0)**

   If yes, from where?

   1. Media/News
   2. Class/School
   3. Movie
   4. Television
   5. Book
   6. Other People
   7. Other – specify

   ____

If you did decide to participate and we had all of this information about your genes…

M. Would you want to know if we find anything that could have health implications? Y(1) / N (0)

N. What would you want to know about it?
   1. Things I could prevent or treat
   2. Things that are very likely to happen.
   3. Things that might affect my children or grandchildren
   4. Anything that might have implications for my health (all of the above)

5. Other - Specify.

____

____

O. How would you like to be told (multiple answers possible)?
   1. Phone
   2. Letter
   3. Email
   4. Sending information through your primary care physician
   5. Through a genetic counselor
   6. Other – specify

      ____

P. What would be the most important things to know?
   Examples of information that could potentially be learned: risks for inherited neurological diseases like Huntington's disease, cancer risk, heart disease, diabetes, Alzheimer's, to list a few.

____

____

Q. What would you do with that information (multiple answers possible)?
   1. Nothing
   2. Talk to doctor
   3. Talk to family members
   4. Talk to a scientist/geneticist

R. Would you change your behavior based on this information?
   1. Yes
   2. Maybe
   3. No
   4. Don't know

   Specify: Why?

   ____

   ____

S. Would your decision about wanting to know this information be influenced by whether or not you can do something about it? For example, a disease that will affect you no matter what, versus a disease that is medically treatable or preventable?
   Y(1) / N(0)

Specify____

____

____

T. Would having this information influence your decision to have children, or, if you already do have children, would it have influenced your decision?

   Y(1) / N (0)

   Specify____

   ____

   ____

Privacy Protection and Research Ethics

U. What do you feel is the researcher's responsibility with regard to this genetic information?

   Specify:

   ____

   ____

   ____

V. Do you feel that someone should be responsible for informing your relatives?

   Y (1) / N (0)

   1. If yes, who? ____
   2. If no, why not? ____

W. Would you consent to other researchers accessing your genetic information for different studies, if it was anonymous?

   Y (1) / N (0)

X. Do you have any concerns about who could access this information?

   Y (1) /N (0) – who?

   Specify:

   ____

   ____

Y. Do you have any concerns about "genetic discrimination," especially when it comes to things like health insurance and employment?

1<br><br>Not at all	2<br><br>A little concerned	3<br><br>Somewhat Concerned	4<br><br>Concerned	5<br><br>Very Concerned

Probably need to give examples here: If you were found to have a genetic predisposition for early-onset Alzheimer's, genetic discrimination could include not being hired for a job or being denied life insurance coverage.

Specify:

____

____

Z. Would you agree to your genetic information being made publicly available, for example, on the internet, if it was anonymous? Why/why not?
Y (1) / (N)
Specify:

____

____

AA. Are you worried that your personal genetic information could be used against you somehow?
Y (1) / N (0)

1<br><br>Not at all	2<br><br>A little concerned	3<br><br>Somewhat Concerned	4<br><br>Concerned	5<br><br>Very Concerned

Specify:

____

____
